# Climate change drives mountain butterflies towards the summits

**DOI:** 10.1038/s41598-021-93826-0

**Published:** 2021-07-13

**Authors:** Dennis Rödder, Thomas Schmitt, Patrick Gros, Werner Ulrich, Jan Christian Habel

**Affiliations:** 1 Zoologisches Forschungsmuseum Alexander Koenig, Adenauerallee 160, 53113 Bonn, Germany; 2 Senckenberg German Entomological Institute, Eberswalder Straße 90, 15374 Müncheberg, Germany; 3 Department of Zoology, Faculty of Natural Sciences I, Institute of Biology, Martin Luther University Halle-Wittenberg, 06099 Halle (Saale), Germany; 4 Entomology and Biogeography, Institute of Biochemistry and Biology, Faculty of Science, University of Potsdam, 14476 Potsdam, Germany; 5 Haus der Natur, Museumsplatz 5, 5020 Salzburg, Austria; 6 Department of Ecology and Biogeography, Nicolaus Copernicus University PL-Toruń, 87-100 Toruń, Poland; 7 Evolutionary Zoology, Department of Biosciences, University of Salzburg, 5020 Salzburg, Austria

**Keywords:** Ecology, Evolution, Zoology, Climate sciences, Ecology, Environmental sciences

## Abstract

Climate change impacts biodiversity and is driving range shifts of species and populations across the globe. To understand the effects of climate warming on biota, long-term observations of the occurrence of species and detailed knowledge on their ecology and life-history is crucial. Mountain species particularly suffer under climate warming and often respond to environmental changes by altitudinal range shifts. We assessed long-term distribution trends of mountain butterflies across the eastern Alps and calculated species’ specific annual range shifts based on field observations and species distribution models, counterbalancing the potential drawbacks of both approaches. We also compiled details on the ecology, behaviour and life-history, and the climate niche of each species assessed. We found that the highest altitudinal maxima were observed recently in the majority of cases, while the lowest altitudes of observations were recorded before 1980. Mobile and generalist species with a broad ecological amplitude tended to move uphill more than specialist and sedentary species. As main drivers we identified climatic conditions and topographic variables, such as insolation and solar irradiation. This study provides important evidence for responses of high mountain taxa to rapid climate change. Our study underlines the advantage of combining historical surveys and museum collection data with cutting-edge analyses.

## Introduction

Habitat destruction and climate change are the main drivers of the global biodiversity crisis^1^. Climatic conditions have considerably changed all over the world^2^, and major parts of Central Europe are characterised by increasingly hot and dry periods in summer^3^. Such changes of climatic conditions modify species community compositions^4^, impacts species interactions^5^, and shape species’ distribution ranges, with shifts towards higher altitudes and latitudes^6,7^. Effects from climate change on biodiversity are particularly visible in mountain regions, where species often occupy specific climatic niches, frequently combined with high ecological specialisation, hence making them highly sensitive to environmental changes^8,9^. Most of these species are highly specialized on specific hostplants and to abiotic conditions (e.g. climatic niche); in addition, they are adapted in their evolution to interact with the phenologies of other taxa. Thus, marginal changes of abiotic and biotic conditions can disturb and interrupt inter-specific interactions^10,11^. Butterflies are particularly sensitive to environmental changes, such as climatic shifts, because many representatives of this group of species are strictly adapted to certain environmental conditions, and their development depends on certain larval food plants and specific microhabitat structures^12^. Therefore, this group is an ideal study system to investigate recent changes due to climate change. Species respond very differently to changes in their environment depending on their niche breadths^13,14^. Studies have shown that species with a broad ecological amplitude can cope significantly better to rapidly occurring environmental changes^15^. In contrast, specialized species that require very specific resources such as habitat structures, a specific climatic niche, or the presence of a particular larval food plant, may be much more negatively affected by environmental changes^16^. Dispersal behaviour also plays a central role: Species with a high degree of mobility can respond much better to environmental changes such as habitat degradation and fragmentation or shifts in climate than species with a low propensity to dispersal, which usually remain in one habitat for many generations^17^. To analyse species’ specific responses on climate change, long-term observations in combination with detailed knowledge on species’ ecology, behaviour and life-history are necessary^18^. Most butterflies are taxonomically and ecologically well understood if compared with other invertebrates^18–21^, and thus provide an excellent model system to analyse potential climate change effects.

In this study, we analyse distributional trends of mountain butterflies (also including one burnet moth species) of the Federal State of Salzburg in northern Austria, Central Europe for the past six decades. Our study region represents a pronounced altitudinal gradient ranging from less than 400 m a.s.l. to almost 4000 m a.s.l. This region is strongly affected by climate change during the past few decades^22^, with well-visible effects on biodiversity, as evident by studies on the alpine vegetation^23^. Extensive records of butterfly observations exist over several decades and have been compiled into a database, including exact date of observation and its exact location and elevation. In addition, detailed information on the species’ ecology, behaviour and life history exist for most butterfly species and were compiled from various literature sources^21,24–26^. Hereby we paid special attention to the ecological specialization as well as the dispersal behaviour of the butterflies. This compilation of information and records forms the basis to test responses of mountain butterflies to climate warming over time. We address the following research questions:

1. Have mountain butterflies shifted towards higher elevations during the past decades?
2. What are the characteristics (dispersal behaviour, ecological specialisation) of these butterfly species that make them either more resilient or sensitive to climate warming?

## Results

### Environmental niches

A species’ niche breadth can be quantified as multidimensional, irregularly shaped volume in the environmental space. Our hypervolume analyses revealed strong differences in realized niche volumina considering both, climate only and climate plus topographic features. Realized climatic niche volumina had the range 5.8–228.8 (mean = 96.1, sd = 53.2, median = 103.9); differences were much more pronounced when topographic variables (i.e. insolation hours and solar irrigation) were included (3.7–25,894.3, mean = 5715.8, sd = 5591.5, median = 3967.7).

### Species distribution modelling

As environmental variables, we generated PCAs based on climate and topographic features. Using climatic data, PC1 is mainly driven by temperature related variables capturing 42.9% of the total variance, PC2 summarizes precipitation related variables (31.8%), and PC3 is related to precipitation extremes (8.4%). The PCA trained with monthly insolation and irradiation revealed three PCs capturing interannual variations (Supplementary Table S2 online).

The overall performance of our SDMs was good to very good (AUC_training_ 0.762–0.980, AUC_test_ 0.755–0.967; Table 1, Fig. 2). Overall, the temperature related PC1 had the highest contribution, followed by PC2, while the topography related PCs had a low contribution in most models (Supplementary Table S3 online, Supplementary Fig. S2 online). More detailed information for each species is given in Appendix S4 (Zenodo link, 10.5281/zenodo.5059786).

**Table 1:** General linear modelling to infer trait specific variation in the temporal shifts in altitude and altitudinal range sizes.

Variable	df	Altitude		z_alt_		z_range_		z_VMR_		z_Skew_	
		partial η^2^	P(F)	partial η^2^	P(F)	partial η^2^	P(F)	partial η^2^	P(F)	partial η^2^	P(F)
Dispersal ability	2	0.10	0.23	0.14	0.14	0.02	0.73	0.15	0.18	0.05	0.60
Specialisation	1	0.02	0.45	0.01	0.64	0.03	0.39	0.10	0.15	0.02	0.50
Endangerment	1	0.01	0.79	< 0.01	0.72	< 0.01	0.91	0.12	0.10	< 0.01	0.83
Altitude	1	–	–	< 0.01	0.84	0.38	< 0.01	0.10	0.15	0.07	0.23
r^2^		0.14	0.39	0.18	0.38	0.39	0.02	0.33	0.12	0.15	0.61

Medium altitude served as covariate.

Given are effect sizes (partial η^2^ and r^2^ values) and parametric significances.

**Figure 1:**
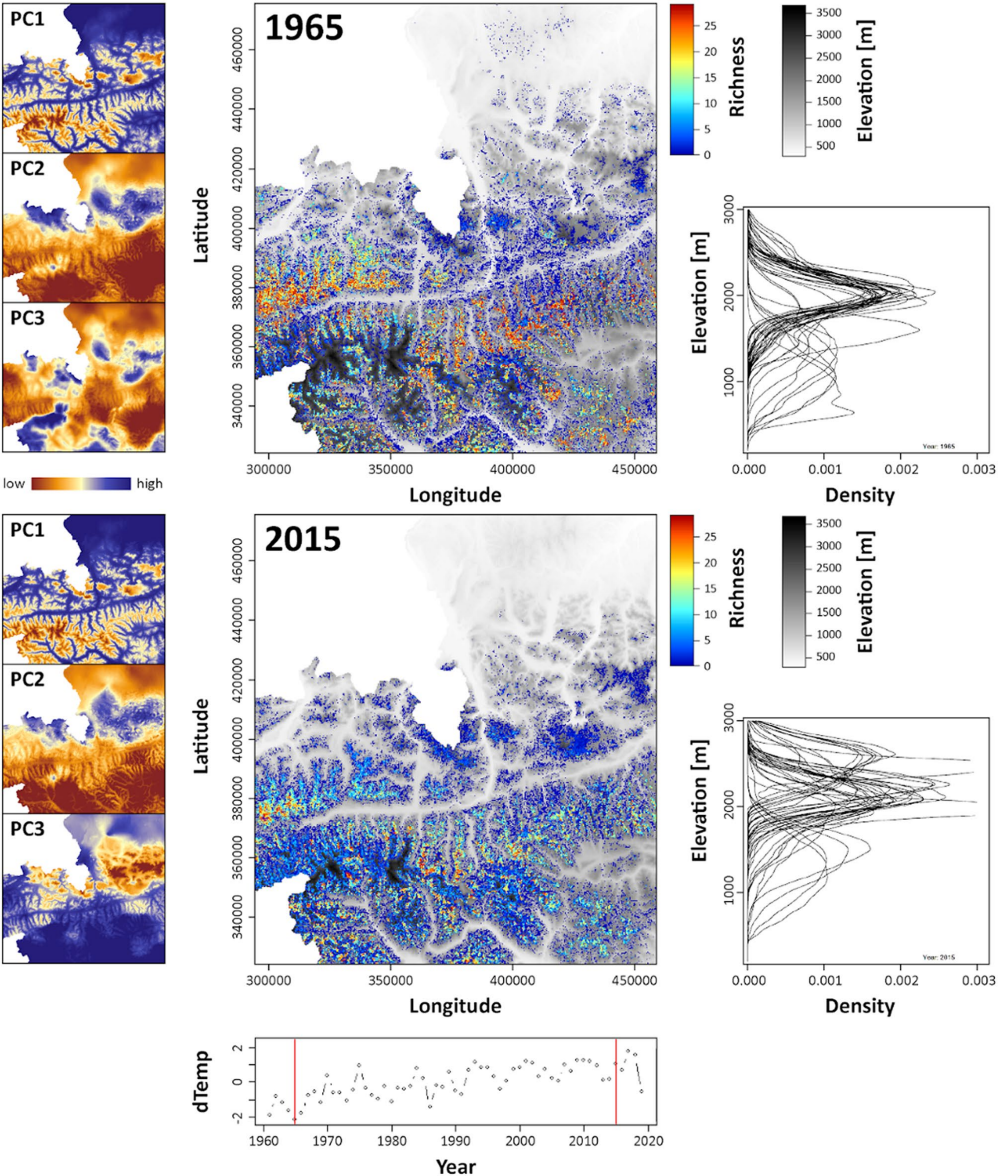
Differences in species richness patterns across the most extreme years 1965 and 2015. PC1-3 refer to the climate related principal components, which were used to train the SDMs (for factor loadings see Supplementary Table S2 online); dTemp refers to the deviation of the annual mean temperature of a specific year relative to the median conditions 1961–2019. Density profiles represent the predicted potential distribution per species in different altitudes. Map projection is Lambert Conformal Conic 2SP.

The potential overall species richness at a given altitude (based on the estimated distributions revealed by their climatic niches) strongly fluctuated throughout the study period. The maximum space covered by the investigated species and hence the highest average species richness throughout the study area was calculated for 1965 with most species finding suitable habitats at comparatively lower altitudes than in 2015, when our models suggested the smallest area occupied by our species (Fig. 1). These differences are due to a pronounced uphill shift in the potential distributions resulting in shrinking range sizes.

### Altitudinal shifts

**Figure 2:**
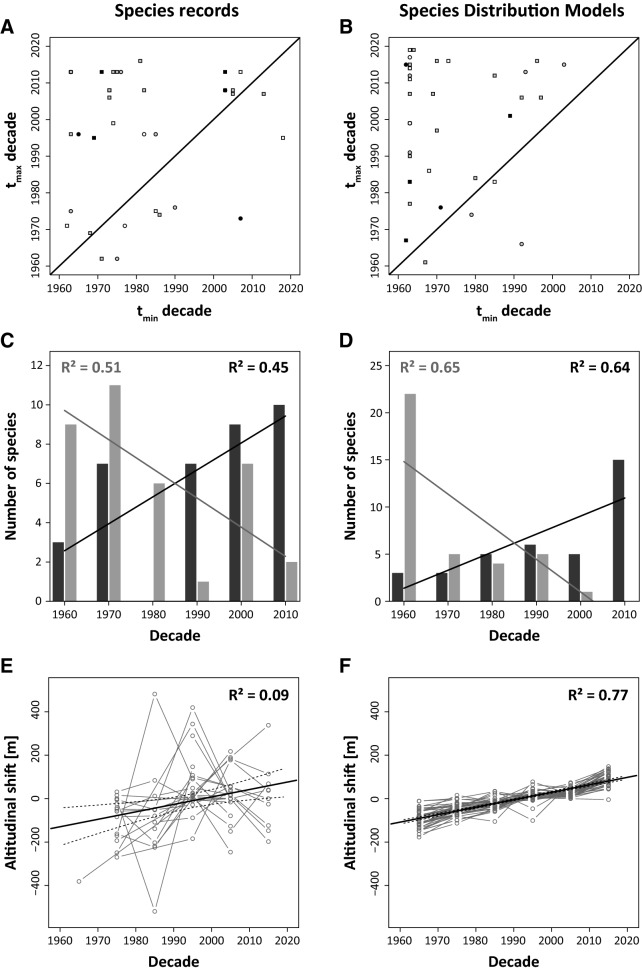
Relationships between the years of minimum and maximum observed (**A**) and modelled (**B**) altitudinal occurrences for 37 mountain butterfly species. The black line denotes the 1:1 relationship. Observed (**C**) and modelled (**D**) numbers of species with their minimum (light grey bars) and maximum (dark grey) observed elevation of occurrence in a focal decade. Median altitudinal shift per decade revealed by the median altitudinal position of each species within a decade minus the median over the complete study period as evident from species records (**E**) and species distribution models (**F**); this analysis was performed for all species. The coefficient of determination R^2^ refer to linear ordinary least squares regression. Parametric significances: **P < 0.01, ***P < 0.001.

27 of the 37 studied species had their highest observed altitude later than their minimum, resulting in an increase of observations at higher altitudes towards more recent years (Fig. 2A); the record at the lowest respective altitude was before 1980 for 21 species. The trait distributions did not significantly change along the altitudinal gradient (Supplementary Table S2 online). The species distribution models returned qualitatively identical results, predicting higher altitudinal occurrences for 32 of the 37 species in more recent years (Fig. 2B). 22 species were predicted to have their minimum altitudinal occurrences before 1970, while 15 species should have had altitudinal maxima during the last decade (Fig. 2). Although there was no significant correlation between the empirical median altitude and decade, we found a respective high correlation when using the predictions of the species distribution models (R^2^ = 0.77; Fig. 2F).

**Figure 3:**
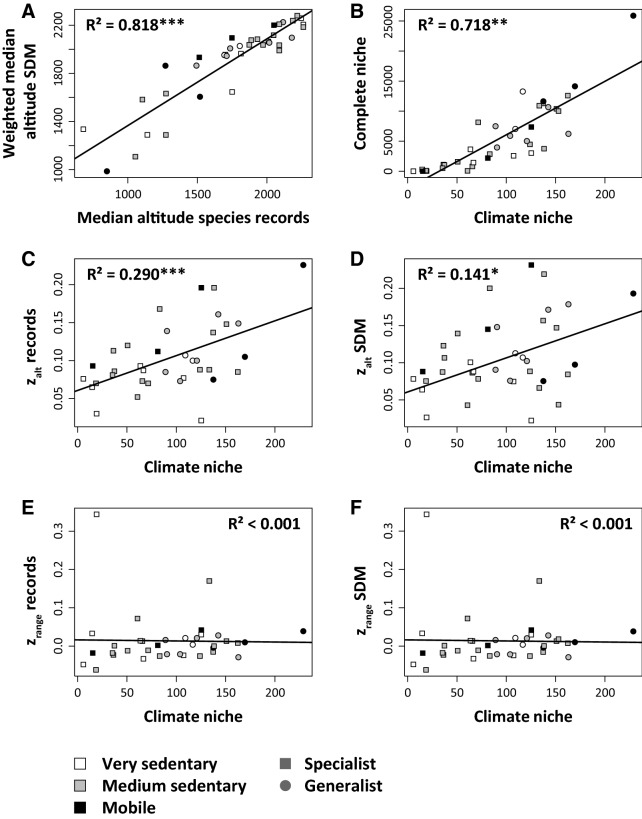
Relationship between median altitude of species records and median altitude derived from the species distribution models weighted by probability (**A**). Relationship between the niche volumes computed based on climatic components only and both climate and topography (**B**). Slopes of altitudinal shift z_alt_ increased with increasing climate niche using both the species records (**C**) and the median altitude per species weighted by SDM probability (**D**). Slopes of range size shifts z_range_ showed no correlation with the climate niche volume using both species records and SDM results (**E, F**). R^2^: coefficients of variation of ordinary least squares regression. Parametric significances: *P < 0.05, **P < 0.01, ***P < 0.001.

The median altitudes obtained from both data sets were highly correlated (R^2^ = 0.82, Fig. 3A), as were the climatic niche volumes and the environmental niche volumes including both climate and topographic variables (R^2^ = 0.72; Fig. 3B). The altitude—study year regression slopes z_alt_ were positive for all species using both approaches and increased with climate niche breadth (Fig. 3C, D) implying that particularly species with large climatic niches (i.e. being generalists in this aspect) tended to move uphill more strongly. This trend did not depend on the distribution of the three traits considered, although sedentary species showed the tendency to have comparatively low z_alt_ values (Fig. 3C, D). This uphill shift was not significantly related to species altitudinal range sizes (Fig. 3E, F).

**Figure 4:**
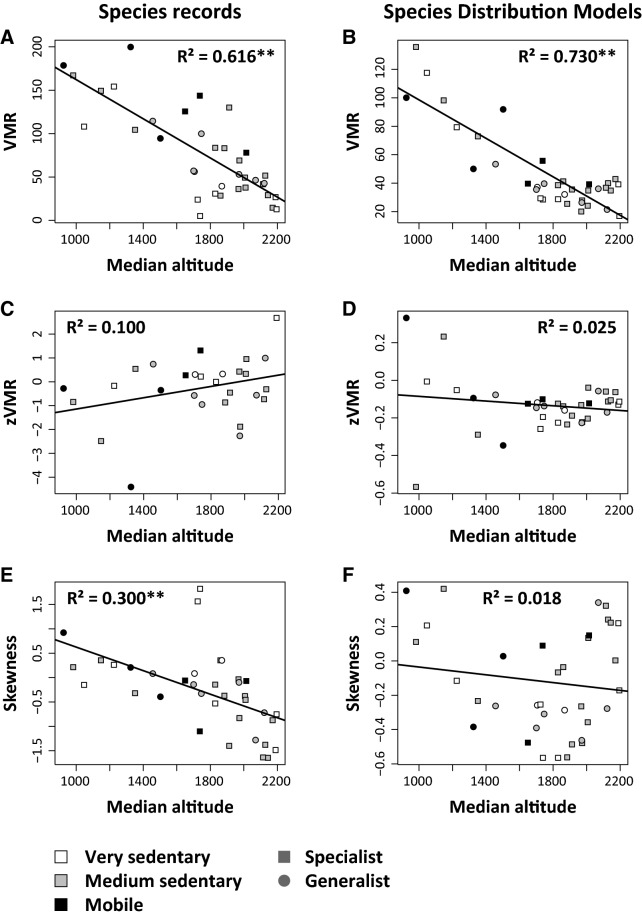
Variance-mean ratios (VMR) of the species altitudinal distribution decreased using both species records and the results from species distribution modelling (SDM) (**A**, **B**). The slopes of the regressions of annual VMR against study year z_VMR_ increased with increasing altitude using species records (**C**) but not using SDM results (**D**). The skewness of the species altitudinal distributions deceased at higher altitudes using both approaches (**E**, **F**). R^2^: coefficients of variation of ordinary least squares regression. Parametric significances: **P < 0.01.

Species altitudinal range sizes as estimated by Variance-Mean Ratios (VMR) decreased with increasing median altitude using both species records and SDM results (Fig. 4A, B). In turn, z_VMR_ slopes increased at higher altitude when using species records (Fig. 4C), but decreased when using SDM results (Fig. 4D). However, in both cases the correlations were rather weak. Species occurring mainly at lower altitudes had right skewed and species of higher altitudes left skewed altitudinal distributions (Fig. 4E, F), but these trends were not sensitively linked to trait distributions (Table 1), although mobile species tended to have comparatively high z_VMR_ values.

## Discussion

### Shifting towards higher elevations

Our results document significant and constant shifts of butterfly distributions in the eastern Alps towards higher elevations during the past six decades. While the highest altitudinal maxima were mostly observed in more recent years, observations at the respective lowest altitudes are restricted to the period prior to 1980. This translates into an average altitudinal distributional shift of the species of more than 300 m uphill within this comparatively short period of time. These findings correspond with previous studies analysing climate change-driven distributional shifts of butterflies, in altitude^7,27–30^, but also in latitude^2,6,31–33^. In general, butterflies are responding highly sensitive and with little time-lag on environmental changes^34^. These prompt responses might be due to their strong and complex links with multiple specific habitat conditions, due to their interactions with other species, but also their comparatively high mobility^12^. Nevertheless, previous studies showed similar range shifts towards higher altitudes in high mountain systems also for other organismal groups, as reported for montane and subalpine bumblebees, with upwards shift of more than 300 m within 33 years^35^, or a shift of almost 500 m of the alpine bumblebee *Bombus alpinus* in the Alps within as little as three decades^36^. Similarly, mountain plant species (the larval food plants of many butterflies) have shifted their distributions considerably towards the summits over time^37^, which might also interrupt interactions among species^38^.

As one of their consequences, these range modifications may result in spatial mismatches between species on the one hand and abiotic and biotic conditions on the other, thus perhaps leading to disturbances in interactions across trophic levels, such as herbivory, parasitism, and mutualistic relations^29^. This is of particular importance as many of the butterfly species analysed in our study depend on specific habitat types and larval food plants. Thus, the fritillary *Euphydryas intermedia* is restricted to open mountain forest habitats offering the only larval food plant, *Lonicera coerulea*; the Small Apollo *Parnassius phoebus* is mostly found in habitats accompanying fast-flowing mountain streams where the most important larval food plant, *Saxifraga aizoides*, is growing at sparsely vegetated spots; or the Sooty Ringlet *Erebia pluto*, one of the butterflies reaching the highest altitudes, that is mostly restricted to the coarse sandy and sparsely vegetated side and end moraines in the glacier fore-fields (PG, pers. observ.). In addition to these examples of extreme habitat specialisation, several other butterfly species considered in our study are (often strictly) mono- or oligophagous, and thus rely on specific larval food plants for their development, such as e.g. *Boloria titania*, *Pyrgus andromedae*, and *P. warrensis*.

For one of these (i.e. the Purple Bog Fritillary *B. titania*), niche modelling showed that global warming might differently impact the ranges of the butterfly and its most important larval food plant, the common bistort *Bistorta officinalis*. The study predicts a reduction of spatial overlap of the climatic niches of both species, maybe even ending up in an almost complete spatial mismatch with detrimental consequences for the butterfly^39^. This might also apply for other mountain butterflies with larval food plant specialisation, as e.g. shown for the Rocky Mountain Apollo *Parnassius smintheus* and its larval food plant, i.e. several species of *Sedum*, mostly *S. lanceolatum*. Filazzola and colleagues predict a potential distribution expansion of this butterfly in the wake of climate warming. However, by integrating the future distributions of the relevant *Sedum* species (under dry-climate scenarios), a relevant reduction of the realised distribution of the butterfly is more likely in the near future^38^. Severe future geographic mismatches between butterflies and their larval food plants become even more likely against the background of diverging response velocities in both groups: The response of plant species to climate warming is rather moderate and slow if compared with flying insects^40^. This may decouple current species-interactions even in case that their theoretic climatic niches do not diverge, what might negatively impact the persistence of mountain insects in the near future.

### Species specific responses

Ecological and behavioural aspects (such as species-specific dispersal abilities) are important factors when studying responses on environmental change. In this context, we observed that species with large climatic niches have moved uphill significantly faster than species with narrow niches; species with low dispersal capacity tended to shift their distributions to higher altitudes less than more mobile species. Thus, in general, the species in our survey with more specialised ecological demands have adapted their altitudinal ranges less to the on-going climatic changes than less specialised species. These findings go in line with other studies showing repeatedly that species’ responses to environmental changes strongly diverge depending on their specific demands, behaviour and plasticity^41^. For example, Filazzola and colleagues^38^ modelled future distributions of five mountain butterfly species in North America and found that only generalist species (relying on a broad variety of different larval food plants) may benefit from distribution range expansions mediated by climate change. In contrast, the Rocky Mountain Apollo *Parnassius smintheus*, a specialist mostly relying on one larval food plant, might lose a significant proportion of its potentially suitable distribution range due to lacking future niche overlaps with this food plant.

Coherence between ecological specialisation and dispersal behaviour has been documented repeatedly for European butterflies showing that high mobility is frequently a generalist trait often associated with broad trophic ranges, long flight periods, and other features facilitating survival in changing environments^42,43^. In consequence, high ecological specialisation paired with poor mobility is increasing the risk of extinction^33,41^, as mobility is often inversely related to local population density^44^, so that little mobile species may need larger habitat areas to survive and are less likely to survive in rapidly changing environments, as demonstrated in our study for their altitudinal flexibility.

For these reasons, species-specific responses to climate change may also result in considerable modifications of community structures and may thus shape future community assemblies. Moving to higher altitudes at different velocities may change community composition even within genera^7,35^. As discussed above, this can disrupt special overlap between herbivores and their food plants, but also may impact interactions among species, such as mutualistic relations between pollinators and flowering plants^45,46^; however, also the formation of new and intact communities is feasible. In this context, it is also important to emphasise that not only pollinators depend from nectar and pollen sources and phytophages from plant biomass, but that many plant species rely on specific pollinators for their successful reproduction^47^. Consequently, these complex dependencies and interactions underline that not only insects might lack resources, but that also new competitive interactions may be formed, among others by lowland species that expand their distributions uphill into the subalpine and alpine zones with their communities, in addition to the climatic components, potentially deteriorating the living conditions of the mountain species by their competition.

### Conclusion

Our study provides strong evidence for altitudinal range shifts of mountain butterflies in the eastern Alps. As these changes differ among species, they might result in profound community modifications with possible effects on species interactions and competition among taxa. Our study hereby underlines the high scientific value of long-term data sets to understand past and predict future range changes and biodiversity trends. Nevertheless, we have to consider the limitations of our data set. Thus, our results show strong fluctuations of records over time what might be due to two possible non-exclusive reasons: Firstly, arthropod populations and thus their detectability fluctuate severely among generations and years, as e.g. shown for our study group, the butterflies^48^. Thus, local populations, in particular at the edge of the species’ altitudinal distribution, can easily fall below the detection limit, which may distort results. Secondly, the assessment of butterflies for the here analysed data set has not been performed according to a standardized protocol. Nevertheless, and despite these drawbacks, our study reveals a clear trend in mountain butterfly distributions—towards higher elevations.

## Methods

### Data set

We exclusively considered mountain butterflies which have only exceptional occurrences below 600 m a.s.l. in temperate latitudes. Furthermore, we only considered species with sufficient data records for Salzburg over time (i.e. 13). The data used for this study are primarily based on the entomological collection of the “Haus der Natur”, museum of natural sciences in Salzburg (https://www.hausdernatur.at/en/). These data were completed by further records of recent butterfly assessments conducted across our study region, as well as by various literature sources. All species records are stored in the biodiversity database of the “Haus der Natur”. In total, we considered 5836 records from 37 species (i.e. 36 butterfly and one burnet moth species). The data points cover a period from 1960 until 2019. We chose this time window as it contained sufficient local records per year and as it is demarking the onset of Eastern Alpine temperature increase captured by our climate data. We used the following information from each single record: Species name, date of observation, exact location of observation (GPS coordinates), and elevation. Two local entomologists (P. Gros and G. Embacher) recently reviewed the quality of the entire data set. Sources used are compiled in Appendix S1. The raw data set is provided in Appendix S2.

For distribution modelling, a total of 4094 records collected between 1961 and 2019 were available (Supplementary Fig. S1 online). The minimum number of records was 13 for *Erebia pluto*, the maximum 438 for *Pieris bryoniae* (mean = 110.6, sd = 195.1, median = 102.0).

### Topographic and climatic data

Climatic data was obtained from the SPARTACUS project^49,50^. For each year from 1961 till 2019, raster layers representing monthly minimum, mean and maximum temperature as well as cumulative precipitation with a spatial resolution of 1 km^2^ were available. These monthly temperature related variables were further downscaled to a spatial resolution of 50 m using a geographically weighted regression (gwr function from the spgwr package for R^51^). As co-variable, a digital elevation model with an original resolution of 10 m available from http://gis.ktn.gv.at/OGD/Geographie_Planung/ogd-10m-at.zip was used, which was developed using airborne laser scans. This step was necessary in order to reduce computation time. As we expect only a weak correlation between elevation and precipitation, these variables were downscaled to the same resolution using a bipolar interpolation available in the raster package for R^52^. We acknowledge that the interpolation approach may lead to uncertainties in climate reconstructions. However, due to the strong correlation between elevation and temperature, we are confident to receive realistic scenarios. Furthermore, as we compare the results of our distribution models with those obtained from the species records themselves, we expect to find similar trends using both approaches.

As the microhabitat features in a mountainous area may strongly depend on quantity and quality of insolation, which strongly dependent on aspect and slope, we computed monthly insolation time and irradiation (W/m^2^ h) using the relevant functions in QGIS 3.10.2. Dimensionality was reduced using a principal component analysis (PCA) resulting in three principal components with eigenvalues > 1. In a second step, bioclimatic variables were computed for each year using the biovars function of the dismo package for R, excluding overly complex variables^53^ (Supplementary Table S2 online). Bioclimatic conditions were extracted at each species record from the respective year of discovery, and a second principal component analysis was trained in order to reduce dimensionality (Supplementary Table S2 online). This PCA model resulted in three PCs with eigenvalues > 1 and was projected onto each time slice, and the distribution models were trained using both the PCs obtained from bioclimatic and topographic information (see below).

### Traits and climatic niches

We divided the species into three basic ecological trait categories, (a) whether they are habitat generalist or specialist (euryocy), (b) whether they are sedentary, intermediate, or mobile (dispersal), and (c) whether they are threatened/vulnerable (endangerment) or least concern according to the Austrian Red List of butterflies^54^. The compiled information is given in Appendix S3.

### Niches and potential distributions

For each species record, we assigned the respective PCA transformed bioclimatic and insolation values based on its spatial position and year of discovery. Niche volumes of each species were computed using multivariate hypervolumes delimited by support vector machines (package hypervolume for R^55^) based on (1) bioclimatic and insolation data and (2) separately for bioclimatic data. Both scores allow the quantification of the degree of niche specialisation for each species, as specialists are expected to occupy smaller realised niche volumes than generalists.

The potential distributions of each species in each year were assessed using Maxent 3.4.0^56–58^ using the swd-data format as described above. Analogously, random background data were generated by extracting the environmental data from 10,000 records randomly sampled for each year (59,000 in total). For each species, we computed 100 Maxent models randomly splitting the records in 80% used for model training and 20% for model testing using the Area Under the Receiver operating characteristic curve (AUC)^59^ using a bootstrap approach. Feature classes were restricted to linear, quadratic and product features as initial tests revealed the most realistic, bell shaped response curves. As output format, the cloglog format was used, and final projections of the models to each time slice were rescaled to a range between 0 and 1 using the average 10% training omission threshold across all replicates, i.e. we reclassified all probabilities of occurrence below this threshold to nodata, subtracted the threshold and divided the maximum probability by the score of each gridcell.

All potential distributions were masked using the relevant land-cover classes, which represent suitable microhabitats according to expert opinions. As high-resolution land cover information we used the Ecosystem Type Map v.3.1^60^ (available from https://www.eea.europa.eu/data-and-maps/data/ecosystem-types-of-europe-1) with a spatial resolution of 100 m. As such data is only available for 2012 and no coherent land cover information is available for other time slices, it was not possible to include habitat availability directly in the SDMs. In order to quantify elevational shifts between each year, we computed a weighted mean based on elevation and probability of occurrence for each grid cell. This estimate may counterbalance the availability and stochastic fluctuation of single species records and hence represents a rather holistic view on the distributional changes.

### Statistics

We use three different approaches to answer our starting questions, each time using the chosen set of 37 species and comparing results obtained from the species records themselves, and the species distribution models. First, we determined for each species the years of maximum (t_max_) and minimum (t_min_) altitude record for both the species records themselves and the weighted medians of the potential distributions (median altitude weighted by probability). According to our first starting question, we expected to obtain higher altitudinal occurrences in more recent years and lower at the beginning of our analysed time frame. While the species records may be affected by some sort of temporal and spatial sampling biases, SDMs may counterbalance a potential sampling bias by providing annual estimates, but on the other hand be also prone to uncertainties due to climate reconstructions. We expected that both approaches are complementary and thus provide strongest evidence if their results are in concert.

Next to minimum and maximum altitudinal occurrences, we also assessed if the median elevation has changed over time. Therefore, we computed the median elevation per decade for both the species records and the SDMs (weighted according to probability) and used simple linear regressions to identify trends over time.

In a second approach, we looked at altitudinal shifts in records and changes in species range sizes along the altitudinal gradient. For each species, we therefore calculated (a) the slope of a linear regression between the year of record and the altitude of the record (z_alt_) and (b) the respective slope for the regression between annual range size and study year (z_range_). Under the assumption that species shifted their occurrences uphill and their range sizes expand towards higher altitudes, we expected to see a prevalence of positive slopes for both regressions.

Third, we inferred whether the species altitudinal distributions are skewed, that is whether only a small part of the population occurred at high altitudes while the majority of individuals of each species remained downhill or vice versa. A simple approach to this question is to again use a linear regression between the study year and the skewness of the altitudinal distribution within each year (z_skew_). To reduce the noise stemming from too low sample sizes when using species records, we calculated the skewness only for study years where a given species occurred in at least five sites of different altitude. The final regression was run over a subset of 28 species, which were recorded in the defined number of sites in at least five different study years. For SDMs we used a skewness weighted by probability in an analogue framework. An uphill shift of parts of the populations should be visible in a decrease of annual skewness values with time. For comparison, we also calculated for each species the total degree of skewness in altitudinal occurrence over all study years. Given that the species distributions are bounded by upper and lower occurrence limits, we expected to see a negative correlation of skewness with the centre of occurrence (approximated by the median) and a negative skewness of uphill and a positive skewness of downhill species.

Finally, we looked at the species variability in altitudinal range sizes over time and calculated for each species the variance-mean ratios (VMR) for each study year and across all study years as a simple quantification of range size:

1 $$ VMR = \frac{{\sigma ^{2} \left( h \right)}}{{\mu \left( h \right)}} $$

where *h* denotes the species occurrence vector and σ^2^ and μ are the respective variance and mean. As before, we calculated for each species the linear regression slopes z_VMR_ between annual VMR values and study years. According to our first research question, we expected to see a temporal increase in altitudinal range size. For statistical inference, we used ordinary least squares linear regression and general linear modelling. To answer our second research question, we used general linear modelling to refer mean altitudinal occurrences, z_alt_, z_range_, z_VMR_, and z_skew_ (response variables) to species dispersal ability as well as the degrees of specialisation and endangerment (predictors).

## Supplementary Information

Supplementary Information 1.

Supplementary Information 2.

Supplementary Information 3.

Supplementary Information 4.
