# Supplementary material for: Climate change drives mountain butterflies towards the summits
Source: Sci Rep. 2021 Jul 13;11:14382. doi: 10.1038/s41598-021-93826-0 (PMC8277792; doi:10.1038/s41598-021-93826-0)

Supplementary Table S1: Overview of data sources considered in our study. Given are number of records, and respective percentages on the total data set. Abbreviations: BioZ: Biologiezentrum, Linz; HdN: Haus der Natur, Salzburg; LMK: Landesmuseum Kärnten, Klagenfurt; NHM: Naturhistorisches Museum, Wien; NPHT: Nationalpark Hohe Tauern; TLMF: Tiroler Landesmuseum Ferdinandeum, Innsbruck; ZSM: Zoologische Staatssammlung, München.

| **Source** |
| --- |
| Data base of PG |
| Lepidoptera collection of the Haus der Natur |
| Data base of GE |
| Data base of ARGE and records of HdN |
| Various records (provided by ZSM, TLMF, BioZ, LMK, NHM) |
| Data of NPHT |
| Data from publications |

Overview of all references used.

Embacher, G. & Gros, P. 2013. Die Schmetterlinge des Salzburger Glocknergebietes (In­sec­ta: Le­pidoptera). – Mit­t. Haus der Natur **21**: 5-24.

Embacher, G., Gros, P., Kurz, M., Kurz M. & Zeller-Lukashort, C. 2011. Die Schmetterlinge des Lan­des Salzburgs: Systematisches Verzeichnis mit Verbreitungsangaben für die geo­lo­gi­sch­en Zonen des Landes (Insecta: Lepidoptera). – Mit­t. Haus der Natur **19**: 5-89.

Gros, P. & Kurz, M. 2013. Die Insektenfauna des Gemeindegebietes Neumarkt am Wal­lersee (Ös­ter­reich, Salzburg): eine bemerkenswerte Vielfalt mit hohem naturschutz­fachlichem Wert. – Sauteria **20**: 107-125.

Gros, P. 2005a. Natura 2000 Gebiet Wallersee-Wengermoor in Salzburg – Schmet­ter­lings­­mo­nitoring nach dem LIFE-Projekt - Erfassung der Anhang II-Arten *Maculinea nau­sithous* (Dunk­ler Wiesenknopf-Ameisenbläuling), *Maculinea teleius* (Heller Wie­sen­knopf-Amei­sen­bläu­ling) und *Euphydryas aurinia* (Abbiß/Skabiosen-Schecken­fal­ter). – Report commissioned by the Nature conservation department of the State of Salzburg.

Gros, P. 2005b. Kartierung der Tagfalter (Lepidoptera: Papilionoidea und Hesperioidea) und der Libellen (Odonata) Im Egelsee-Moor im Gemeindegebiet Puch bei Hallein (Salzburg) mit Vorschlägen zum geeigneten Management. – Report commissioned by the Nature conservation department of the State of Salzburg.

Gros, P. 2006a. Natura 2000 Gebiet Bluntautal in Salzburg - Kartierung der Tagfalter (Le­pi­dop­tera: Papilionoidea und Hesperioidea) mit Vorschlägen zum geeigneten Management. – Report commissioned by the Nature conservation department of the State of Salzburg.

Gros, P. 2006b. Kartierung der Tagfalter (Lepidoptera: Papilionoidea und Hesperioidea) und der Libellen (Odonata) Im Mandling-Moor im Gemeindegebiet Radstadt (Salz­burg) mit Vor­schlägen zum geeigneten Management. – Report commissioned by the Nature conservation department of the State of Salzburg.

Gros, P. 2007. Erhebung der Tagfalter (Lepidoptera: Papilionoidea und Hesperioidea) und Li­bellen (Odonata) im Ursprunger Moor (Gemeindegebiete Elixhausen und See­kir­chen am Wal­lersee, Salzburg). – Report commissioned by the Austrian Association for the Protection of Nature.

Gros, P. 2008a. EU-relevante Tagfalterarten (EWG 1992/43, Annex II/IV) im EU-Schutz­ge­biet Untersberg-Vorland: LIFE-Projekt/ Bestandserhebungen 2008. – Report commissioned by the Nature conservation department of the State of Salzburg.

Gros, P. 2008b. Erstnachweis des Hellen Wiesenknopf-Ameisen-Bläulings *Maculinea te­leius* (Berg­strässer, 1779) aus dem Salzburger Ennstal sowie weitere bemerkens­wer­te Funde die­ser Art im Bundesland Salzburg (Lepidoptera: Lycaenidae). – Beiträge zur En­to­mo­fau­nis­tik **9**: 123-128.

Gros, P. 2010. EU-relevante Schmetterlingsarten (EWG 1992/43, Annex II/IV) im Bun­­des­land Salzburg. Zusammenfassung des aktuellen Kenntnisstandes. – Report commissioned by the Nature conservation department of the State of Salzburg.

Gros, P. 2012a. Monitoring EU-relevanter Tagfalterarten (EWG 1992/43, Annex II/IV) im EU-Schutz­­gebiet Un­ters­berg-Vorland: Ergebnisse für das Jahr 2012. – Report commissioned by the Nature conservation department of the State of Salzburg.

Gros, P. 2012b. Erhebung der Schmetterlingsfauna in einer Siedlung in Guggenthal bei Koppl, am östlichen Rand der Stadt Salzburg (Österreich): Erste Ergebnisse (Insecta: Le­pi­doptera). – Mit­t. Haus der Natur **20**: 38-56.

Gros, P. 2015a. Die Gefährdungssituation des Blauschillernden Feuerfalters, *Lycaena helle* (De­nis & Schiffermüller, 1775), einer Art der Anhänge II & IV der FFH-Richtlinie, im Bun­des­land Salzburg, Österreich: Erste Ergebnisse (Lepidoptera: Lycaenidae). – Mit­t. Haus der Natur **22**: 63-70.

Gros, P. 2015b. Natura 2000 Gebiet Wallersee-Wengermoor Erfassung der Anhang II-Arten *Ma­culinea nausithous* (Dunkler Wiesenknopf-Ameisenbläuling) und *M. teleius* (Heller Wie­sen­knopf-Ameisenbläuling) in den Flächen des Naturschutzbunds. – Report commissioned by the Austrian Association for the Protection of Nature.

Gros, P. 2016a. Für das Pinzgauer Salzachtal neue oder bemerkenswerte Schmet­ter­lings­ar­t­en (Land Salz­burg, Bezirk Zell am See) (Le­pidoptera: Yponomeutidae, Gly­phip­terigidae, Tor­tricidae, Zygaeni­dae, Hesperiidae, Nymphalidae, Lycaenidae, Crambidae, Sphingidae, Geo­metridae, Noctuidae). – Mit­t. Haus der Natur **23**: 25-28.

Gros, P. 2016b. Erhebung der Tagfalter- und Libellenfauna im Rahmen des Projektes „Land­schafts­pflegeplan Kalkmergelbruch Gutrathberg 2015-2017“. – Report commissioned by the Institute for Ecology in Salzburg (IfÖ).

Gros, P. 2018. Schigebiet Schmitten – Zell am See - Erfassung der Tagfalterfauna unter­schied­lich bewirtschafteter Schipisten im Gebiet der Schmittenhöhe. – Report commissioned by the Institute for Ecology in Salzburg (IfÖ).

Gros, P., Dolek, M., Strausz, M. & Wittmann, H. 2015b. Erfassung des Gelbringfalters (*Lopinga a­chine*) im Saalachtal zwischen Unken und Saalfelden. – Report commissioned by the Salzburg AG.

Gros, P., Illich, I., Ramsauer, N., Stöhr, O. & Stüber, E. 2011. Lebensräume, Flora und Fau­­na des Tennengebirge-Südabfalles im oberen Lammertal. Eine Grundlagener­he­bung (2010-2011). – Report commissioned by Peter Kaindl.

Supplementary Table S2: PCA summaries based on climate only (PC1-3) and topographic features (radPC1-3). Abbreviations are bio1 = Annual Mean Temperature; bio5 = Max Temperature of Warmest Month; bio6 = Min Temperature of Coldest Month; bio7 = Temperature Annual Range; bio8 = Mean Temperature of Wettest Quarter; bio9 = Mean Temperature of Driest Quarter; bio10 = Mean Temperature of Warmest Quarter; bio11 = Mean Temperature of Coldest Quarter; bio12 = Annual Precipitation; bio13 = Precipitation of Wettest Month; bio14 = Precipitation of Driest Month; bio16 = Precipitation of Wettest Quarter; bio17 = Precipitation of Driest Quarter; bio18 = Precipitation of Warmest Quarter; bio19 = Precipitation of Coldest Quarter; insolX = Insolation Month X; irradX = Irradiation Month X.

| **Variable** | **PC1** | **PC2** | **PC3** |  | **Variable** | **radPC1** | **radPC2** | **radPC3** |
| --- | --- | --- | --- | --- | --- | --- | --- | --- |
| **bio1** | 0.89 | 0.41 | 0.03 |  | **insol1** | 0.83 | 0.06 | 0.47 |
| **bio5** | 0.87 | 0.38 | -0.01 |  | **insol2** | 0.88 | -0.06 | 0.36 |
| **bio6** | 0.66 | 0.26 | 0.26 |  | **insol3** | 0.87 | -0.33 | 0.15 |
| **bio8** | 0.80 | 0.38 | 0.04 |  | **insol4** | 0.80 | -0.55 | -0.01 |
| **bio9** | 0.72 | 0.43 | -0.13 |  | **insol5** | 0.71 | -0.67 | -0.02 |
| **bio10** | 0.88 | 0.38 | -0.01 |  | **insol6** | 0.66 | -0.70 | -0.01 |
| **bio11** | 0.81 | 0.42 | 0.18 |  | **insol7** | 0.69 | -0.69 | -0.02 |
| **bio12** | -0.54 | 0.79 | 0.07 |  | **insol8** | 0.75 | -0.62 | -0.02 |
| **bio13** | -0.53 | 0.64 | 0.39 |  | **insol9** | 0.83 | -0.46 | 0.06 |
| **bio14** | -0.25 | 0.61 | -0.59 |  | **insol10** | 0.88 | -0.19 | 0.25 |
| **bio16** | -0.57 | 0.69 | 0.39 |  | **insol11** | 0.86 | 0.02 | 0.44 |
| **bio17** | -0.36 | 0.74 | -0.42 |  | **insol12** | 0.81 | 0.07 | 0.48 |
| **bio18** | -0.51 | 0.65 | 0.35 |  | **irad1** | 0.73 | 0.56 | 0.26 |
| **bio19** | -0.31 | 0.74 | -0.31 |  | **irad2** | 0.81 | 0.53 | 0.07 |
|  |  |  |  |  | **irad3** | 0.85 | 0.42 | -0.24 |
|  |  |  |  |  | **irad4** | 0.86 | 0.27 | -0.40 |
|  |  |  |  |  | **irad5** | 0.88 | 0.07 | -0.43 |
|  |  |  |  |  | **irad6** | 0.88 | -0.08 | -0.41 |
|  |  |  |  |  | **irad7** | 0.88 | -0.02 | -0.42 |
|  |  |  |  |  | **irad8** | 0.88 | 0.17 | -0.43 |
|  |  |  |  |  | **irad9** | 0.85 | 0.35 | -0.34 |
|  |  |  |  |  | **irad10** | 0.84 | 0.48 | -0.09 |
|  |  |  |  |  | **irad11** | 0.78 | 0.55 | 0.19 |
|  |  |  |  |  | **irad12** | 0.71 | 0.56 | 0.28 |
|  |  |  |  |  |  |  |  |  |
| **Eigenvalues** | 6.01 | 4.45 | 1.17 |  |  | 16.00 | 4.33 | 2.11 |
| **Explained Variance** | 42.92 | 31.77 | 8.37 |  |  | 66.68 | 18.03 | 8.79 |

Supplementary Table S3: Performance, variable contributions and presence/absence threshold of the species distribution models.

|  | **Mean** | **Median** | **SD** | **Min** | **Max** |
| --- | --- | --- | --- | --- | --- |
| **Training.AUC** | 0.889 | 0.889 | 0.061 | 0.762 | 0.980 |
| **Test.AUC** | 0.877 | 0.873 | 0.062 | 0.755 | 0.976 |
| **PC1.contribution** | 38.7 | 37.6 | 13.8 | 15.2 | 66.9 |
| **PC2.contribution** | 17.6 | 16.8 | 7.5 | 6.4 | 38.4 |
| **PC3.contribution** | 5.9 | 4.3 | 4.6 | 0.6 | 21.7 |
| **radPC1.contribution** | 13.3 | 10.6 | 7.2 | 5.7 | 36.5 |
| **radPC2.contribution** | 11.9 | 10.7 | 6.1 | 4.2 | 33.6 |
| **radPC3.contribution** | 12.6 | 9.6 | 9.8 | 2.8 | 48.1 |
| **10.percentile.training.presence.Cloglog.threshold** | 0.24 | 0.24 | 0.07 | 0.08 | 0.39 |

Supplementary Figure S1: Our study region, the Federal State of Salzburg in northern Austria, is characterized by severe altitudinal gradients along the eastern edge of the Alps (large map, color ramp from black [lowland] to white [high altitudes]). Species records are indicated as white dots. This figure was constructed with the open access software and open data of QGis (https://www.qgis.org/de/site/).


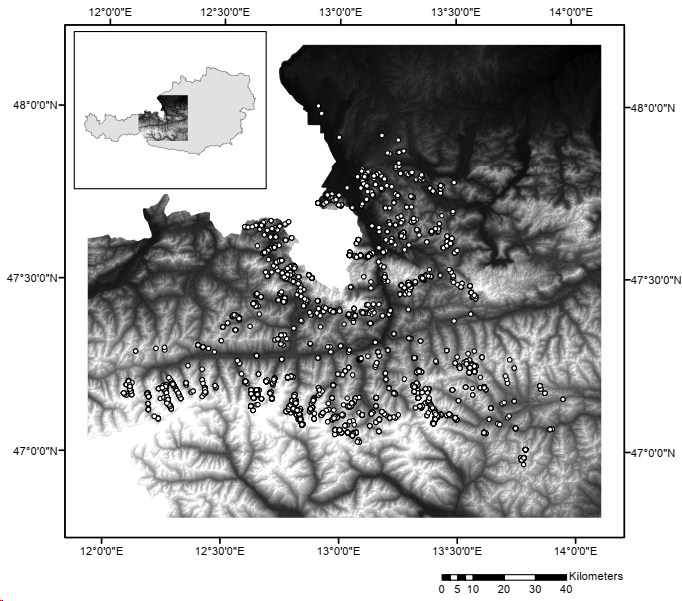


Supplementary Figure S2: Summary of key parameters for species distribution modelling of 37 butterfly species. Number of training and test samples (a), model performance in terms of training and test AUC (b), and variable contributions (c). Median per species of each 100 replicates were used to generate the boxplots.


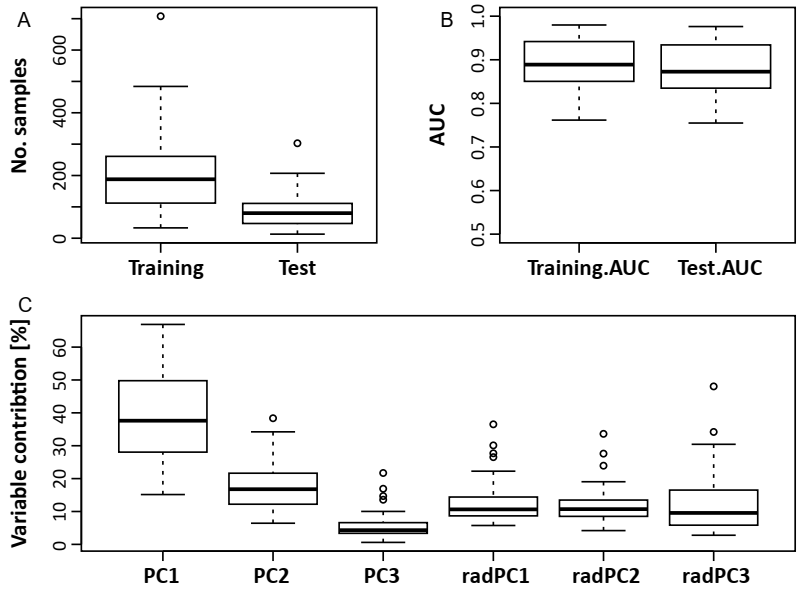

Supplement: Supplementary file 1 — Supplementary Information 1. [file 41598_2021_93826_MOESM1_ESM.docx]
