# Supplementary material for: Climate change drives mountain butterflies towards the summits
Source: Sci Rep. 2021 Jul 13;11:14382. doi: 10.1038/s41598-021-93826-0 (PMC8277792; doi:10.1038/s41598-021-93826-0)
Supplement: Supplementary file 4 — Supplementary Information 4. [file 41598_2021_93826_MOESM4_ESM.docx]

Appendix S4 to Zenodo (<https://zenodo.org/record/5059786#.YN62l-gzaM8>). The doi is  10.5281/zenodo.5059786.
